# A probabilistic coevolutionary biclustering algorithm for discovering coherent patterns in gene expression dataset

**DOI:** 10.1186/1471-2105-13-S17-S12

**Published:** 2012-12-07

**Authors:** Je-Gun Joung, Soo-Jin Kim, Soo-Yong Shin, Byoung-Tak Zhang

**Affiliations:** 1 Seoul National University Biomedical Informatics (SNUBI), Seoul 110-799, Korea; 2 Systems Biomedical Informatics National Core Research Center, Seoul 110-799, Korea; 3 Institute of Endemic Diseases, Seoul National University College of Medicine, Seoul 110-799, Korea; 4 Interdisciplinary Program in Bioinformatics, Seoul National University, Seoul, 151-742, Korea; 5 Department of Clinical Epidemiology & Biostatistics, Asan Medical Center, Seoul, 138-736, Korea; 6 University of Ulsan College of Medicine, Seoul, 138-736, Korea; 7 School of Computer Science and Engineering, Seoul National University, Seoul, 151-744, Korea

## Abstract

**Background:**

Biclustering has been utilized to find functionally important patterns in biological problem. Here a bicluster is a submatrix that consists of a subset of rows and a subset of columns in a matrix, and contains homogeneous patterns. The problem of finding biclusters is still challengeable due to computational complex trying to capture patterns from two-dimensional features.

**Results:**

We propose a Probabilistic COevolutionary Biclustering Algorithm (PCOBA) that can cluster the rows and columns in a matrix simultaneously by utilizing a dynamic adaptation of multiple species and adopting probabilistic learning. In biclustering problems, a coevolutionary search is suitable since it can optimize interdependent subcomponents formed of rows and columns. Furthermore, acquiring statistical information on two populations using probabilistic learning can improve the ability of search towards the optimum value. We evaluated the performance of PCOBA on synthetic dataset and yeast expression profiles. The results demonstrated that PCOBA outperformed previous evolutionary computation methods as well as other biclustering methods.

**Conclusions:**

Our approach for searching particular biological patterns could be valuable for systematically understanding functional relationships between genes and other biological components at a genome-wide level.

## Background

Since many biological data could be represented as a two-dimensional matrix, it is important to find the hidden structure contained within such a structure. Here, the hidden structure can mean the clusters embedded in the subspace in a high-dimensional dataset [1]. The problem of finding these structures can be solved using biclustering, which is also known as coclustering or block clustering [2-5]. A bicluster is a submatrix that consists of a subset of the rows (e.g., genes) and a subset of columns (e.g., conditions) in the matrix. The purpose of biclustering is to find the submatrix that consists of homogeneous elements in rows, columns, or both. Biclustering has been applied to diverse areas such as frequent itemsets, information retrieval, and gene expression analysis [4,6].

Biclustering has been intensively studied in molecular biology research, as the expression levels of thousands of genes can be measured experimentally using microarrays [7]. DNA microarray data are represented as a matrix of expression levels of genes under different conditions corresponding to a set of rows and a set of columns. Here, the conditions usually include the environment, diseases, and tissues. The biclustering algorithm tries to find a subset of the genes representing similar behavior under multiple conditions. The biclustering problem is known as an NP-hard combinatorial problem [2].

Biclustering problems are more complex than one-way clustering problems, because of the coupled landscapes of their search space. Biclustering problems may reflect the issues encountered in evolving the interdependent subcomponents considered in coevolutionary learning. In biclustering problems, the rows and columns of a matrix can be considered as interdependent subcomponents. If a biclustering algorithm is permitted to interact between these subcomponents, then it can search efficiently in a coupled landscape. For example, Potter and De Jong suggested the potential problem-solving capability of cooperative coevolutionary systems [8,9] and following study of Zaritsky and Sipper presented good results for the *Shortest Common Superstring *(*SCS*) problem, using a cooperative coevolutionary algorithm [10].

Here, we propose a Probabilistic COevolutionary Biclustering Algorithm (PCOBA) to find functional groups of genes and corresponding conditions from microarray datasets. It is based on the concept of coevolutionary learning and probabilistic searching. The most distinctive idea of PCOBA is that it decomposes the entire search space into subcomponents to discover hidden patterns in the matrix. In this algorithm, two populations, corresponding to a subset of rows and a subset of columns, are maintained. Coevolutionary learning evolves the two different populations within the context of each other [11-13]. PCOBA guides these populations towards the minimum of the objective function representing the quality of the biclusters through cooperation between two populations.

When applied to synthetic datasets and the microarray data of yeast, the results demonstrate PCOBA incorporating probabilistic searching improves its ability of finding biclusters. The resulting patterns are well enriched to known annotations that are consistent with biological knowledge. Our approach for searching important biological patterns could be utilized to find the uncovered relationships between genes and other biological components at a genome-wide level.

## Methods

### Biclustering of microarray data

In gene expression data, it is defined as a subset of genes and a subset of the conditions. Let *G *= {*g*_1_, *g*_2_, ..., *g_N_*} be a set of genes and *C *= {*c*_1_, *c*_2_, ..., *c_M_*} be a set of conditions, such as different tissue samples. The data can be represented as an *N* × *M *matrix with real values, denoted as *E*. Here each entry, *e_ij_*, in *E *indicates the expression level of a gene, *g_i_*, under a specific condition, *c_j_*.

Let *I *be the set of row indices belonging to a row cluster, and *J *be the set of column indices belonging to a column cluster, where *I* ⊆ {1,...,*N*} and *J* ⊆ {1,...,*M*}. Thus, a bicluster is a submatrix, *B *= (*I, J*), |*I*|≤*N *and |*J*|≤*M*, where *I *and *J *indicate the set of genes (rows) and conditions (columns), respectively. The volume of a bicluster, (*I, J*), is defined as the number of entries, *e_ij_, i* ∈ *I *and *j* ∈ *J*.

**Figure 1:**
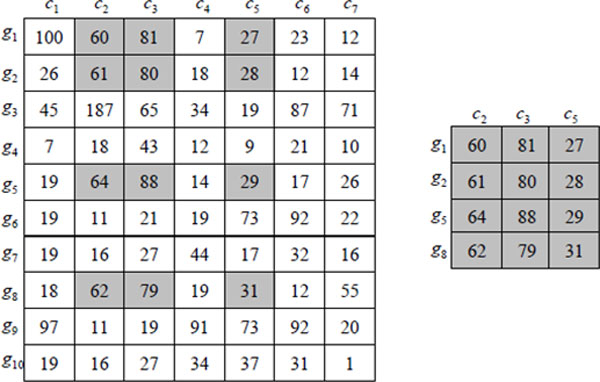
**Example of biclustering**. The rows represent genes and the columns represent conditions. All the elements in the bicluster are highlighted in gray.

An example of a data matrix, *E*, and a bicluster, (*I, J*), is shown in Figure 1. In this example, the bicluster could be *B *= ({*g*_1_, *g*_2_, *g*_5_, *g*_8_}, {*c*_2_, *c*_3_, *c*_5_}) in the expression matrix.

To find a bicluster with the required quality, we first consider the mean squared residue (MSR), as proposed by Cheng and Church [2]. This is the squared mean residue of all the elements in a submatrix, (*I, J*),

$$H_{IJ}=\frac{1}{\left|I||J\right|}\underset{i\in I,j\in J}{\sum }h_{ij}^{2},$$

where *h_ij _*is the residue of an element *e_ij _*in the bicluster determined by index sets *I *and *J*. The residue of an element *e_ij _*is defined as

$$h_{ij}=e_{ij}-e_{iJ}-e_{Ij}+e_{IJ}.$$

The residue is the difference between the actual value of an entry and the expectation value of an entry. As the residue of an entry decreases, its coherence in its rows and columns gets stronger. Here

$$e_{iJ}=\frac{\underset{j\in J}{\sum }e_{ij}}{\left|J\right|},e_{Ij}=\frac{\underset{i\in I}{\sum }e_{ij}}{\left|I\right|},e_{IJ}=\frac{\underset{i\in I,j\in J}{\sum }e_{ij}}{\left|I||J\right|},$$

where e_*iJ*_ indicates the mean of the entries in row *i*, of which column indices are in *J*. e_*Ij*_ indicates the mean of the entries in column *j*, of which row indices are in *I*. e_*IJ*_ is the mean of all the entries in the submatrix consisting of *I *and *J*.

If only MSR is applied to measure the quality of a bicluster, then the trivial biclusters, such as biclusters showing no fluctuation in expression level can be found. The raw variance reject any trivial biclusters as follows,

$$V_{IJ}=\frac{1}{\left|I||J\right|}\underset{i\in I,j\in J}{\sum }{\left(e_{ij}-e_{iJ}\right)}^{2}.$$

By adding this term to an objective function, it is possible to detect fluctuations in the gene expression levels under some conditions or samples.

To find a bicluster, we present the objective function to minimize it by employing some characteristics.

• Minimizing the mean squared residue, *H_IJ_*. If a mean squared residue of a specific bicluster has lower than a parameter value, *δ*, then its bicluster is denoted as *δ*-bicluster.

• Maximizing variance, coupled with highly coherent biclusters.

• Maximizing volume, which means a large number of genes and conditions.

### Probabilistic coevolutionary biclustering

Various attempts have been made to find biclusters in microarray data [2,14-16]. Several evolutionary algorithms for biclustering have also been proposed. Bleuler et al., introduced an evolutionary algorithm coupled with previous biclustering algorithms [17]. Mitra et al., proposed a multi-objective evolutionary biclustering algorithm incorporating local search strategies [18]. They demonstrated that evolutionary algorithms can successfully improve the quality of biclusters. The search strategy of our algorithm is different from those using conventional operators. Our algorithm utilizes the global statistical information of two cooperative populations so that its ability to search biclusters is more effective. The key idea is that the algorithm coevolves two populations for a gene set and a condition set, as the one is adapted cooperatively to the other.

**Figure 2:**
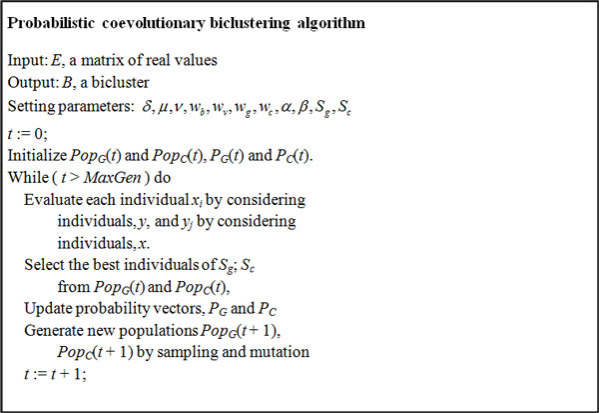
**Probabilistic coevolutionary biclustering algorithm**. *Pop*(*G*) is a population for gene set and *Pop*(*C*) is that for condition set. Individuals, *x_i _*and *y_j _*are evaluated and the bests are selected. The probability vectors of two populations, *P_G _*and *P_C _*are updated and new populations are generated by sampling and mutation in each iteration. Each parameter indicates: *δ *(cutoff of residue score); *μ *and *ν *(initial size of gene and condition population); *w_b _*and *w_v _*(parameters controlling the variance and volume); *w_g _*and *w_c _*(parameters keeping a balance between the genes and condition); *α *and *β *(parameters controlling update of probability); *S_g _*(best individuals in genes); *S_c _*(best individuals in conditions, respectively).

The pseudo code of PCOBA is shown in Figure 2. Each individual in the population of the gene (or condition) sets is encoded using binary vectors that represent a subset selected from the gene (or condition) set. The fitness of each individual is determined by the degree of cooperation between the selected one and individuals of the other population. The two populations are updated using statistical information extracted from the previous populations and mutation operator. After setting the parameters, the initial populations, PopG of size *μ *and PopC of size *ν*, are created randomly from the gene sets and the condition sets. Each individual is evaluated by measuring the fitness functions. Then, sets of the best individuals, Sg and Sc are selected from the current populations. Next, the probabilities PG and PC are updated using the update rule based on the distribution of the selected individuals. Populations of the next generation are generated based on the current updated probability vectors.

#### Coevolutionary optimization

The population of the gene set, Pop_*G*_ and that of the condition set, Pop_*C*_ consist of {*x*_1_, *x*_2_, ..., *x_μ_*} and {*y*_1_, *y*_2_, ..., *y_ν_*}, respectively. Here, each individual *x_i _*is encoded by a binary string, $\left(x_{i}^{1},x_{i}^{2},\ldots ,x_{i}^{N}\right)\in {\left\{0,1\right\}}^{N}$, that represents the presence of several genes among a set of genes, {*g*_1_, *g*_2_, ..., *g_N_*}.

In addition, *y_j _*for a given set of conditions is encoded in the same way as x_*i*_ is. Therefore, the total search space is Ω = {0, 1}*^N ^*+ {0, 1}*^M^*. A bicluster, (*I, J*), is an index with a value = 1 in (*x_i_, y_j_*) pair, *i *= 1,...,*N *and *j *= 1,...,*M*.

#### Fitness evaluation

The score function is designed to measure the quality of a bicluster [19]. The minimum score denotes the best quality that should have a low mean squared residue, high variance, and large volume. This bicluster may satisfy that the expression patterns of many genes are similar in many different conditions.

It consists of three terms, as follows

$$Score\left(x_{i},y_{j}\right)=RES_{IJ}+VAR_{IJ}+VOL_{IJ}$$

First, *RES *indicates the residue score, and is measured by

$$RES_{IJ}=\left\{\begin{array}{cc} \frac{H_{IJ}}{\delta } & if\;H_{IJ}>\delta \\ 1 & else. \end{array}\right)$$

If H_*IJ*_ is greater than *δ*, then *RES *reflects the mean squared residue, else it is set as a constant. Here *δ *is predefined by user. When *RES *is a constant, the fitness can concentrate more on the variance and volume terms.

Second, the variance term is

$$VAR_{IJ}=w_{b}∙\frac{1}{V_{IJ}}.$$

Here, w_*b*_ is a parameter controlling the variance term among all the terms.

Finally, the volume term is

$$VOL_{IJ}=w_{v}∙\left(w_{g}∙\frac{\left|I\right|}{N}+w_{c}∙\frac{\left|I\right|}{M}\right).$$

Here, *w_v _*is a control parameter used to set an importance to the volume term among the terms. The terms *w_g _*and *w_c _*are weight parameters used to keep a balance between the genes and conditions.

The fitness of each individual is measured from the scores defined in previous equation, and is obtained when it forms the complete solution (i.e., the bicluster) with an individual of the other species. An individual of the other species is referred to as a "collaborator''. The fitness of an individual *x_i _*is

$$F\left(x_{i}\right)=\text{min}Score\left(x_{i},y_{i}\right),\;j=1,...,M.$$

and that of *y_j _*is

$$F\left(y_{i}\right)=\text{min}Score\left(x_{i},y_{i}\right),\;i=1,...,N.$$

The minimum score determines the fitness of each individual when it is combined with individuals from the other population. In terms of coevolution, individuals are adapted cooperatively to the other population.

Here, it may be not necessary to evaluate the fitness to calculate the scores between all the *x *and *y *pairs. If the algorithm calculates all the scores of the pairs to select the best collaborator, then the evaluation cost will be high. To reduce the evaluation cost, we applied the following strategy. The algorithm selects the number of *R, R*≤*M*, randomly for each *y_j_*, and then it calculates their scores. Thus, the total number of evaluations is reduced by *R*⋅*ν *in each generation. Since this strategy can affect performance, appropriate *R *value (> = 10% of *M*) should be carefully chosen.

#### Probabilistic update of a population

The next population is generated by sampling with a probabilistic distribution and mutation operator. While the probabilistic update of populations utilizes statistical information from the previous generation, the mutation operator involves utilizing the location information in the solution space. A strategy related to the combination of an EDA and a conventional operator [20,21] can improve the performance with regards to the optimality and convergence of conventional genetic algorithms.

In probabilistic learning, two populations, *Pop*(*G*) and *Pop*(*C*), maintain probability vectors, $P_{G}=\left(p_{g}^{1},\;p_{g}^{2},...,p_{g}^{N}\right)$ for the gene set *Pop*(*G*) and $P_{C}=\left(p_{c}^{1},\;p_{c}^{2},...,p_{c}^{M}\right)$ for the condition set *Pop*(*C*), respectively. The initial vector has a uniform distribution. The probabilities are updated using the following equations,

$$\begin{array}{c} p_{g}^{i}=\left(1-\alpha \right)\cdot p_{g}^{i}+\alpha \cdot \frac{\overset{S_{g}}{\underset{k=1}{\sum }}x_{k}^{i}}{S_{g}}\text{and}\\ p_{c}^{j}=\left(1-\beta \right)\cdot p_{c}^{j}+\beta \cdot \frac{\overset{S_{c}}{\underset{k=1}{\sum }}y_{k}^{j}}{Sc}, \end{array}$$

where *α* ∈ (0, 1) and *β* ∈ (0, 1) are the parameters for controlling the updates. This updating rule is similar to the population-based incremental learning (PBIL) algorithm [22]. In each generation, two sets of best individuals, *S_g _*and *S_c _*are selected based on the fitness, and each probability is updated based on the fraction of the number, ones in the selected individuals. This probabilistic model for generating the next population is relatively simple.

We applied an additional mutation operator to generate offspring because it could be helpful for increasing the diversity of population. The number of individuals selected for mutation was different from *S_g _*and *S_c_*, and was set to maintain a sufficient selection pressure. Thus, half of the population size was generated by a probability distribution, and the other half was generated by a mutation operator.

### Other evolutionary algorithms

Here, we describe three different types of algorithm for comparison with other evolutionary algorithms.

#### Genetic algorithm (GA)

The genotype of a bicluster is a continuous bit string, $\left(x_{i}^{1},x_{i}^{2},\ldots ,x_{i}^{N},y_{i}^{1},y_{i}^{2},\ldots ,y_{i}^{M}\right)$. Here, reproduction and mutation are used as genetic operators. A crossover operator was not applied in this study, since a crossover operator tends to form biclusters with a high volume, which interrupts to obtain good solutions. In reproduction, individuals were selected using a proportional selection. The population size was 100, and the mutation rate was set to 0.05.

#### Coevolutionary genetic algorithm (CGA)

Unlike a conventional genetic algorithm, the genotype of a bicluster is not a continuous bit string. The genotype of a CGA is separated into two parts. The genetic operators are the same as the genetic algorithm, and the method of evolution is the same as the PCOBA.

#### Estimation of the distribution algorithm (EDA)

The encoding of individuals here was the same as in the genetic algorithm. However, the next population was generated from a probability vector based on the PBIL algorithm and a mutation, such as the PCOBA. The probability vector was $\left(p_{g}^{1},p_{g}^{2},...,p_{g}^{N},p_{c}^{1},p_{c}^{2},...,p_{c}^{M}\right)$.

## Results

### Experimental data preparation and parameter setting

We performed experiments to show the performance of PCOBA, including both synthetic datasets and a yeast gene expression dataset. The synthetic datasets are *E_a_, E_b_*, and *E_c_*, which were noisy matrices like gene expression datasets. They had embedded homogenous block structures like submatrices coupled between genes and conditions. Their matrices were filled with random values ranging from 0 to 500, and then a fixed number of clusters were embedded. First, we examined whether the proposed PCOBA could find the single homogeneous block structure from *E_a _*which embeds only one bicluster. *E_a _*is the noisy matrix of 100 rows × 20 columns with single structure of (16 × 9).

Furthermore, we studied if PCOBA were able to find the multiple homogeneous block structures in *E_b _*embedding multiple biclusters. Although the volumes of datasets were relatively small, it could be difficult to find biclusters if a block is very homogeneous. Therefore, to make these kinds of matrices, we designed a block structure embedding more homogeneous blocks. *E_b _*contains three different structures (16 rows × 9 columns, 10 rows × 5 columns, and 10 rows × 10 columns) in the noisy matrix of 100 × 20. These structures were less than *δ *= 20. Here, *δ *is the threshold of residue score and lower score means high quality biclusters.

The *E_c _*was used to examine the ability of finding a bicluster from a higher dimensional dataset. Real datasets, such as gene expression data, are composed of large dimensional matrices. In general, if the dimension of a matrix gets larger, then the volume of the biclusters is increased. In addition, the matrix contains a higher number of biclusters. We designed the synthetic dataset, *E_c_*, considering these conditions. *E_c _*is a 1,500 × 30 matrix that contained three 100 × 15 structures. All the block structures were less than *δ *= 300.

The real datasets were gene expression profiles of yeast microarrays. Typically, a microarray experiment assesses the expression of a large number of genes under various conditions. These conditions may be a time series during a biological process, or a collection of different tissue samples, e.g., normal versus tumor tissues. The performance of our proposed algorithm was measured using the cell cycle expression data of a yeast *Saccharomyces cerevisiae *that was obtained from Tavazoie et al., [23]. The matrix dataset contains expression levels of 2,884 genes (rows) under 17 conditions (columns). In this matrix, missing values were replaced by sampled random numbers from a uniform distribution between 0 and 600.

The experimental parameters are listed in Table 1 (see Methods section). In the case of a dataset with large dimensions, we gave much weight to the volume term. In addition, we gave much weight to the gene rather than condition of the volume term. The terms *S_g _*and *S_c _*corresponded to 20% of the population size. The selection ratio of the best individuals for mutation was set to 50%, and the mutation rate was set to 0.01. These values were chosen empirically as the result of multiple runs. For example, when *α *and *β *were small, the algorithm showed a stable performance on the whole.

**Table 1:** Parameter setting of PCOBA

Parameter	Description	Artificial dataset	Real dataset
***μ***	Pop. size for genes	100 (1000)	1000
***ν***	Pop. size for conditions.	50	100
***MaxGen***	Maximum generation	100 (200)	500
***δ***	Cutoff of residue score	20 (300)	250
***w_b _***	Controlling the variance	0.5	0.5
***w_v _***	Controlling the volume	10 (30)	30
***w_g _***	Keeping a balance between	0.9 (0.8)	0.8
***w_c _***	gene and condition	0.1 (0.2)	0.2
***α, β***	Controlling update of probabilities.	0.2, 0.2	0.2, 0.2
***S_g_, S_c_***	Size of best individuals in genes and conditions	20, 10 (200, 10)	200, 20

() corresponds to *E*_c _dataset.

### Searching biclusters using the PCOBA

**Figure 3:**
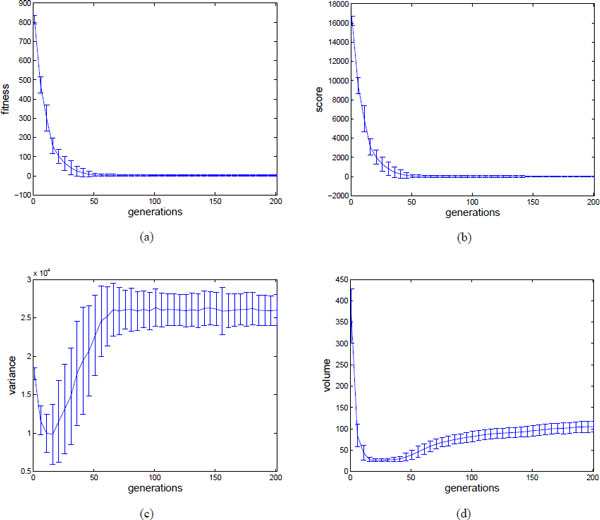
**Simulation results of PCOBA using the synthetic dataset, *E_a_***. (a) A plot showing the fitness over generations. (b) The mean residue score at each generation. (c) The variance versus generation is shown. (d) A plot showing the change in volume. These plots show the average and variance of 100 runs.

We observed the characteristics on optimization, while PCOBA was running with parameter setting of Table 1. Figure 3 shows the simulation results of PCOBA using the synthetic dataset, *E_a_*. The fitness decreased rapidly during the first 20 generations. The score curve was similar to the fitness curve. This means that PCOBA concentrated on the mean residue score by about the 20th generation. In Figure 3(c), the variance is seen to increase after about 20 generations. Although the variance fluctuated over the generation in each run, the general trend is revealed by plotting averages of variants. The volume curve is shown in Figure 3(d). After the algorithm reached a minimum volume, then the volume size increased continually. Here, though we demonstrated an optimization process with *E_a _*dataset, PCOBA also tends to be similar characteristics using other dataset.

### Comparison with other evolutionary algorithms

In this section, we present a comparison of the performance between PCOBA and other evolutionary algorithms. The purpose of this comparison was to analyze the effect of coevolution, an estimation of the distribution, and finally the potential synergy of two different strategies.

We applied four different algorithms, including Genetic Algorithm (GA), Coevolutionary Genetic Algorithm (CGA) [11], Estimation of the Distribution Algorithm (EDA) [24] and the proposed PCOBA, to the synthetic datasets. For a fair comparison, the number of evaluations was the same for all algorithms. First, the runs for the *E_a _*and *E_b _*datasets were terminated after the following iterations. For GA and EDA, the number of iterations was set to 100 populations × 1,000 generations. For CGA and PCOBA, it was set to 100 populations × 10 selected genes × 100 generations. Here 10 selected genes correspond to *R *value (see Methods section) to reduce the evaluation cost. Second, the number of iterations for the *E_c _*dataset was set to 1,000 populations × 1,000 populations for GA and EDA. For CGA and PCOBA, it was set to 1,000 populations × 10 selected genes × 100 generations.

A comparison of the performance between PCOBA and other evolutionary algorithms is shown in Table 2. The results contain the averages and standard deviations after 100 runs. The fitness was mostly affected by the residue score. For *E_a _*and *E_b _*datasets, the residue scores of PCOBA outperformed the other three algorithms. Our algorithm could find a bicluster including coherent elements better than a conventional evolutionary algorithm, such as the simple GA, could, whereas GA often failed to find a homogenous block structure. The higher standard deviation of the scores and fitness was interpreted as an open failure. Although CGA and EDA showed better residue scores than GA did, they were not superior to PCOBA. EDA tended to improve the volume score, whereas CGA tended to focus on the residue score. This result may indicate that PCOBA takes advantage of both algorithms. When *E_b _*formed multimodal landscapes, our analysis tried to find a single local optimum less than *δ*. In the *E_b _*dataset embedding multiple homogenous blocks, the fitness values of all algorithms were better than those using the *E_a _*dataset.

**Table 2:** Comparison of the performance of PCOBA and other evolutionary algorithms.

Datasets	Algorithms	Avg. Fitness	Avg. Residue	Avg. Variance	Avg. Volume
***E_a_***	GA	11.96 ± 16.32	203.51 ± 323.67	19745 ± 9587.70	105.28 ± 54.28
	CGA	3.90 ± 6.99	36.63 ± 140.32	21220 ± 7202	72.39 ± 20.11
	EDA	5.80 ± 11.14	81.84 ± 220.84	23527 ± 6719.4	127.48 ± 21.64
	PCOBA	**1.88 **± 0.06	0.05 ± 0.00	26254 ± 833.22	104.90 ± 8.49

***E_b_***	GA	5.59 ± 10.16	76.67 ± 201.51	18570 ± 7496.3	107.17 ± 38.87
	CGA	3.05 ± 5.02	20.03 ± 100.81	22489 ± 6876.7	75.49 ± 18.99
	EDA	5.12 ± 8.28	67.63 ± 163.60	20862 ± 6834.7	112.36 ± 44.52
	PCOBA	**2.03 **± 1.35	2.74 ± 26.88	25199 ± 3295.9	99.66 ± 16.92

***E_c_***	GA	2.21 ± 0.02	262.63 ± 9.05	3807.20 ± 1068	470.96 ± 18.90
	CGA	2.20 ± 0.03	263.09 ± 7.55	3229.40 ± 1160.4	443.00 ± 19.07
	EDA	2.22 ± 0.05	263.94 ± 6.96	2359.70 ± 228.74	450.83 ± 50.57
	PCOBA	**1.94 **± 0.05	265.01 ± 4.63	2473.50 ± 176.1	562.63 ± 47.43

Mean and standard deviation values after 100 independent runs are shown.

The lower score means the expression values in cluster are more similar.

Usually, real datasets, such as gene expression data, have large dimensions and contain multiple homogenous blocks, and it is difficult to obtain good solutions using a real dataset. Thus, *E_c _*was utilized as an alternative dataset to evaluate the performance considering the scalability in the dataset size. All the algorithms found scores less than *δ*. The average scores of the three algorithms were little different. However, PCOBA had a high value for the volume term.

### Comparison with other biclustering algorithms

We compared the performance with previous Cheng and Church (CC) and the Order Preserving Submatrix (OPSM) biclustering algorithms using the cell cycle expression data of a yeast *Saccharomyces cerevisiae*. The CC algorithm was proposed by Cheng and Church [2] and employs the heuristic in a relaxed "greedy" search. We set the parameter of the CC algorithm, *δ*, with the identical value to our parameter. The OPSM was introduced by Ben-Dor et al., [25]. It was designed to discover biclusters exhibiting coherent behavior in the columns. Thus, this algorithm focuses on the relative order of the columns.

The performance of the three algorithms is presented in Table 3. All the average and standard deviation values are the result of the ten best biclusters after one run. The residue score of our algorithm was similar to that of the CC algorithm. The average of residue score was less than 220. The average variance of PCOBA was marginally better than that of the CC algorithm. Although the OPSM algorithm yielded high-variance results, its residue score was inferior to those of PCOBA and the CC algorithm. As the OPSM algorithm induces a high variance, then it was easier to show poorer residue scores. This may be due to the characteristic of the OPSM algorithm that focuses on coherent behavior to find biclusters. In respect to the volume, the average volumes of the three algorithms were similar. However, the CC and the OPSM algorithms tended to find larger gene sets. Among all the biclusters they found, the volume of only one bicluster dominated the others. The biclusters found by PCOBA were not larger than the volume size, 200, but PCOBA balanced suitably the three terms as a whole, in that it outperformed other algorithms.

**Table 3:** Performance between PCOBA and other biclustering algorithm.

	PCOBA	CC	OPSM
**Avg. Residue**	219.15 ± 1.14	221.40 ± 8.99	447.72 ± 88.36
**Avg. Variance**	412.11 ± 17.62	404.67 ± 134.26	1224.89 ± 415.95
**Avg. Volume**	1321.30 ± 102.82	1369.18 ± 366.90	1365.40 ± 1642.85
**Avg. Num. (Genes)**	92.40 ± 1.64	98.54 ± 21.89	265.10 ± 412.22
**Avg. Num. (Conditions)**	14.30 ± 0.48	12.18 ± 2.37	8.50 ± 3.02

Mean and standard deviation values of the ten best biclusters after single run are shown.

The lower score means the expression values in cluster are more similar.

### Functional analysis of the discovered clusters by PCOBA

To validate the discovered biclusters, we analysed the functional correlations between clustered genes by Protein Interaction Network Analysis (PINA) [26] for yeast dataset. We show two biclusters with more biological significance in this study. Table S1 (Additional File 1) presents the identified two biclusters with most enriched GO biological process terms and KEEG pathways (*p*-value < 0.01). In particular, 'cell cycle' is exactly assigned as an enriched pathway in Cluster I, of which members are highly modulated by protein interaction. 'metabolic process' related terms are enriched in Cluster II. It has been known that metabolism of methionine has been associated with cell cycle progression [27]. These properties confirm the biological relevance of the identified biclusters.

## Conclusions

We have proposed the biclustering algorithm (PCOBA) that can cluster the rows and columns in a two-dimensional matrix simultaneously, based on coevolutionary searching. PCOBA can be considered to be a synergistic optimization technique that combines a coevolutionary search with a population-based probabilistic search. In particular, it is a novel algorithm that can obtain highly correlated patterns from variables of a two-way problem in a dataset having a matrix form. In detail, it could be an efficient procedure to discover coherent patterns, since our algorithm tries to decompose a task using coevolutionary searching, and utilizes former global information in a complex problem of a large-scale matrix. The performance of the proposed PCOBA was tested using synthetic datasets. Our algorithm outperformed conventional evolutionary computing methods including genetic algorithm, coevolutionary genetic algorithm, and estimation of distribution algorithm. In addition, the results from yeast expression datasets showed that our method can offer biclusters of higher quality in regards to coherent patterns. Our proposed method provides substantial guidance for the development of algorithms for finding hidden patterns from datasets in a matrix form that are generated in various research fields, including biology.

## Competing interests

The authors declare that they have no competing interests.

## Authors' contributions

JGJ implemented the method and wrote the manuscript. SJK analyzed the data and wrote the manuscript. SYS wrote the manuscript. BTZ supervised the study.

## Supplementary Material

Additional file 1 **Table S1 - Enriched interactome modules from yeast modules by PINA**. Click here for file

## References

[B1] Yang J Wang W Wang H Yu P δ-Cluster: capturing subspace correlation in a large data set Proceedings of the 18th International Conference on Data Engineering 2002 (ICDE 2002) 517 528

[B2] Cheng Y Church G Biclustering of expression data Proceedings of International Society for Computational Biology 2000 (ISMB 2000) 93 103 10977070

[B3] Gupta R Rao N Kumar V Discovery of error-tolerant biclusters from noisy gene expression data BMC Bioinformatics 2011 12 Suppl 12 S1 10.1186/1471-2105-12-S12-S1 22168285 PMC3247082

[B4] Liu J Li Z Hu X Chen Y Park E Dynamic biclustering of microarray data by multi-objective immune optimization BMC Genomics 2011 12 Suppl 2 S11 10.1186/1471-2164-12-S2-S11 21989068 PMC3194232

[B5] Smet R Marchal K An ensemble biclustering approach for querying gene expression compendia with experimental lists Bioinformatics 2011 27 14 1948 1956 10.1093/bioinformatics/btr307 21593133

[B6] Dhillon IS Mallela S Modha DS Information theoretic coclustering Proceedings of the 9th International Conference on Knowledge Discovery and Data Mining 2003 (KDD 2003) 89 98

[B7] Madeira SC Oliveira AL Biclustering algorithms for biological data analysis: a survey IEEE/ACM Transactions on Computational Biology and Bioinformatics 2004 1 1 24 45 10.1109/TCBB.2004.2 17048406

[B8] Potter MA De Jong KA A cooperative coevolutionary approach to function optimization Proceedings of the Third Conference on Parallel Problem Solving from Nature 1994 (PPSN 1994) 249 257

[B9] Potter MA De Jong KA Cooperative coevolution: an architecture for evolving coadapted subcomponents Evolutionary Computation 2000 8 1 9 10.1162/106365600568086 10753229

[B10] Zaritsky A Sipper M Coevolving solutions to the shortest common superstring problem BioSystems 2004 76 209 216 10.1016/j.biosystems.2004.05.013 15351144

[B11] Hillis DW Co-evolving parasites improve simulated evolution in an optimization procedure Physica D 1990 42 228 234 10.1016/0167-2789(90)90076-2

[B12] Axelrod R Davis L The evolution of strategies in the iterated prisoner's dilemma Genetic Algorithms and Simulated Annealing 1987 32 41

[B13] Barricelli NA Numerical testing of evolution theories, part I: theoretical introduction and basic tests Acta Biotheoretica 1962 16 69 98 10.1007/BF01556771

[B14] Yang J Wang W Wang H Yu P Enhanced biclustering on expression data Proceedings of the third IEEE Conference on Bioinformatics and Bioengineering 2003 (BIBE 2033) 321 327

[B15] Wu CJ Kasif S GEMS: a web server for biclustering analysis of expression data Nucleic Acids Research 2005 33 W596 W599 10.1093/nar/gki469 15980544 PMC1160230

[B16] Prelic A Bleuler S Zimmermann P Wille A Buhlmann P Gruissem W Hennig L Thiele L Zitzler E A systematic comparison and evaluation of biclustering methods for gene expression data Bioinformatics 2006 22 9 1122 1129 10.1093/bioinformatics/btl060 16500941

[B17] Bleuler S Prelić A Zitzler E An EA framework for biclustering of gene expression data Proceedings of Congress on Evolutionary Computation 2004 (CEC2004) 166 173

[B18] Mitra S Banka H Pal SK A MOE framework for biclustering of microarray data Proceedings of the 18th International Conference on Pattern Recognition 2006 (ICPR'06) 1154 1157

[B19] Divina F Aguilar-Ruiz J Biclustering of expression data with evolutionary computation IEEE Transactions on Knowledge & Data Engineering 2006 18 5 590 602

[B20] Pena JM Robles V Larranaga P Herves V Rosales F Perez MS GA-EDA: Hybrid evolutionary algorithm using genetic and estimation of distribution algorithms Proceedings of 17th Int. Conf. Ind. & Eng. Appl. Artif. Intell. & Expert Syst. 2004 361 371

[B21] Zhang Q Sun J Tsang E An evolutionary algorithm with guided mutation for the maximum clique problem IEEE transaction on Evolutionaly Computation 2005 9 2 192 200 10.1109/TEVC.2004.840835

[B22] Baluja S Population-based incremental learning: a method for integrating genetic search based function optimization and competitive learning School of Comput. Sci., Carnegie Mellon Univ., Pittsburgh, PA, Tech. Rep. CMU-CS-94-163 1994

[B23] Tavazoie S Hughes J Campbell M Cho R Church G Systematic determination of genetic network architecture Nature Genetics 1999 22 281 285 10.1038/10343 10391217

[B24] Pelikan M Goldberg DE Lobo F A survey of optimization by building and using probabilistic models Computational Optimization and Applications 2002 21 1 5 20 10.1023/A:1013500812258

[B25] Ben-Dor A Chor B Karp R Yakhini Z Discovering local structure in gene expression data: the order-preserving submatrix problem J Comput Biol 2003 10 373 384 10.1089/10665270360688075 12935334

[B26] Cowley M Pinese M Kassahn K Waddell N Pearson J Grimmond S Biankin A Hautaniemi S Wu J PINA v2.0: mining interactome modules Nucleic Acids Research 2012 40 D862 865 10.1093/nar/gkr967 22067443 PMC3244997

[B27] Dummitt B Micka WS Chang YH N-Terminal methionine removal and methionine metabolism in Saccharomyces cerevisiae Journal of Cellular Biochemistry 2003 89 964 974 10.1002/jcb.10566 12874831

