# Supplementary material for: A probabilistic coevolutionary biclustering algorithm for discovering coherent patterns in gene expression dataset
Source: BMC Bioinformatics. 2012 Dec 7;13(Suppl 17):S12. doi: 10.1186/1471-2105-13-S17-S12 (PMC3521386; doi:10.1186/1471-2105-13-S17-S12)
Supplement: Additional file 1 — Table S1 - Enriched interactome modules from yeast modules by PINA. [file 1471-2105-13-S17-S12-S1.doc]

## Table S1 - Enriched Interactome Modules from Yeast Modules by PINA

| **Cluster** | **Module Networks** | **Annotation Summary** | **p-value** (< 0.01) |
| --- | --- | --- | --- |
| **I** | **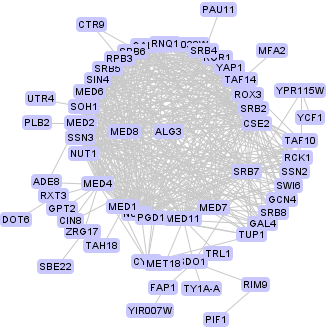** | **GO (Biological Process)**  - transcription from RNA polymerase II promoter [GO:0006366]  - transcription [GO:0006350]  - transcription, DNA-dependent [GO:0006351]  **KEGG Pathway**  - Basal transcription factors [KEGG:03022]  - Glycerophospholipid metabolism [KEGG:00564]  - **Cell cycle - yeast** [KEGG:04111] | 4.30E-3 |
| **II** | **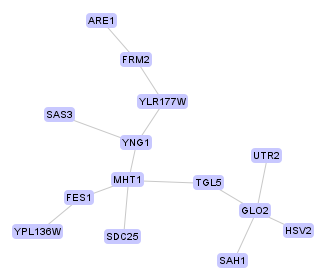** | **GO (Biological Process)**  - methionine metabolic process [GO:0006555]  - histone acetylation [GO:0016573]  - internal protein amino acid acetylation [GO:0006475]  **KEGG Pathway**  - Steroid biosynthesis [KEGG:00100]  - Cysteine and methionine metabolism [KEGG:00270]  - Pyruvate metabolism [KEGG:00620] | 1.60E-3 |
